# Cortical Thinning in the Medial Temporal Lobe and Precuneus Is Related to Cognitive Deficits in Patients With Subcortical Ischemic Vascular Disease

**DOI:** 10.3389/fnagi.2020.614833

**Published:** 2021-02-17

**Authors:** Li Chen, Jiarui Song, Runtian Cheng, Kangcheng Wang, Xiaoshuang Liu, Miao He, Tianyou Luo

**Affiliations:** ^1^Department of Radiology, Affiliated Hospital of North Sichuan Medical College, Nanchong, China; ^2^Department of Radiology, The First Affiliated Hospital of Chongqing Medical University, Chongqing, China; ^3^School of Psychology, Shandong Normal University, Jinan, China

**Keywords:** cognition, cerebral small vessel disease, cortical thickness, magnetic resonance imaging, memory

## Abstract

Subcortical ischemic vascular disease (SIVD) is a major cause of vascular cognitive impairment (CI) and features extensive atrophy in the cerebral cortex. We aimed to test the hypothesis that cognitive deficits in SIVD are linked to decreased cortical thickness in specific brain regions, which may constitute neuroimaging biomarkers of CI. Sixty-seven SIVD patients without (SIVD-NC, *n* = 35) and with (SIVD-CI, *n* = 32) CI and a group of healthy controls (HCs, *n* = 36) underwent structural magnetic resonance imaging (MRI) and cognitive functional assessments. FreeSurfer was used to preprocess structural MRI data and to calculate and compare cortical thickness. The correlation between cortical thickness and cognitive scores was examined in SIVD patients. Significantly altered cortical thickness in the bilateral insula, middle and inferior temporal lobes, precuneus, and medial temporal lobe (MTL) was identified among the three groups (*p* < 0.05, Monte Carlo simulation corrected). *Post hoc* results showed significantly decreased thickness in the bilateral insula and temporal lobe in SIVD-NC and SIVD-CI patients compared with HCs. However, the areas with reduced cortical thickness were larger in SIVD-CI than SIVD-NC patients. SIVD-CI patients had significantly reduced thickness in the bilateral precuneus and left MTL (Bonferroni corrected) compared with SIVD-NC patients when we extracted the mean thickness for each region of interest. In SIVD patients, the thicknesses of the left MTL and bilateral precuneus were positively correlated with immediate recall in the memory test. SIVD might lead to extensive cerebral cortical atrophy, while atrophy in the MTL and precuneus might be associated with memory deficits.

## Introduction

Cerebral small vessel disease refers to a heterogeneous group of pathological disorders that affect the small vessels of the brain and are an important cause of cognitive impairment (CI) (Ter Telgte et al., 2018). Subcortical ischemic vascular disease (SIVD) is the most common small vessel disease and is characterized by extensive cerebral white matter lesions and lacunar infarcts in deep gray and white matter (Roman et al., 2002). SIVD is present to some extent in most individuals aged 60 years or older (de Leeuw et al., 2001) and is a major cause of vascular CI and dementia (METACOHORTS Consortium, 2016; Chen L. et al., 2019). The prevalence increases with age, affecting approximately 5% of people aged 50 years to almost 100% of people older than 90 years (Cannistraro et al., 2019). Half of the patients with a first-ever lacunar infarct of subcortical vascular features have mild cognitive impairment (MCI), and its presence may be a predictor of subcortical vascular dementia in the medium-long term (Grau-Olivares et al., 2007; Jacova et al., 2012). Therefore, neuroimaging markers to identify CI in SIVD patients must be established.

Imaging evidence reveals that reduced brain volume, medial temporal lobe (MTL) atrophy and cortical thinning are related to cognitive dysfunction in both neurodegenerative and cerebrovascular diseases (Chen et al., 2016; Hase et al., 2018; Ter Telgte et al., 2018; Marquez and Yassa, 2019). Brain atrophy is an important predictor of cognitive decline and has been shown to mediate the relationship between the presence of white matter hyperintensities (WMHs) and cognitive decline in individuals (Schmidt et al., 2005; Rossi et al., 2006; Wen et al., 2006; Godin et al., 2009; Jokinen et al., 2012; Raji et al., 2012; Knopman et al., 2015; Tuladhar et al., 2015). WMHs and infarcts can affect the cortex via disruption of white matter tracts (Righart et al., 2013; Duering et al., 2015; Chen L. et al., 2019). A previous study showed that patients with vascular MCI exhibited significantly reduced gray matter volume in regions including the bilateral dorsolateral prefrontal cortex (DLPFC), the orbital portion of the superior frontal gyrus (SFG), the left supplemental motor area and the bilateral posterior cingulate cortex (PCC). However, the cognitive symptoms of SIVD can range from asymptomatic radiological marker occurrence to different degrees of cognitive decline, including MCI and dementia (Chen X. et al., 2019). It is unclear whether there are specific atrophy areas in the cortex related to CI in patients with SIVD and whether there is a difference in cortical atrophy between SIVD without and with cognitive dysfunction.

The primary aim of this work was to investigate the differences in cortical thickness between healthy controls and SIVD patients, including those with normal cognition and CI, using cortical thickness analysis. We hypothesized that cognitive deficits in SIVD are linked to decreased cortical thickness in specific brain regions, which may define neuroimaging biomarkers of cognitive decline.

## Materials and Methods

### Participants

The present study was approved by the ethics committee of our hospital. All subjects provided written informed consent after a total explanation of the procedure involved. In total, 80 patients with SIVD and 40 healthy controls (HC group) were recruited in this study. SIVD was diagnosed according to the following criteria (Roman et al., 2002): (1) WMHs: extensive hyperintensities in periventricular and deep white matter in T2-weighted images; extending caps (measured parallel to the ventricle) >10 mm or an irregular halo >10 mm with broad, irregular margins and extending into deep white matter, and/or diffusely confluent hyperintensities (width >25 mm, irregular margins) or extensive white matter alterations (diffuse hyperintensity without borders); or (2) lacunar infarcts (LI): LI in the subcortex (including deep gray matter and white matter) and a lesion diameter >3 mm but <15 mm.

Patients with SIVD were divided into two groups: those without cognitive deficits (SIVD-NC group) and those with CI (SIVD-CI group). The inclusion criteria for the SIVD-NC group were as follows: (1) fulfillment of the diagnostic criteria for SIVD; (2) no complaints of CI recently and normal daily life activities; (3) Mini-Mental State Examination (MMSE) score ≥27; and (4) Clinical Dementia Rating Scale (CDR) score = 0. The inclusion criteria for the SIVD-CI group were as follows: (1) met the diagnostic criteria of SIVD; (2) participants or their caregivers complained that they had experienced cognitive decline in at least one cognitive domain; (3) MMSE score <26; (4) did not meet the Diagnostic and Statistical Manual of Mental Disorders, fifth edition (DSM-V) criteria for dementia; and (5) CDR score = 0.5.

Exclusion criteria for all participations included a history of craniocerebral trauma, psychiatric or neurological disease, other preexisting brain lesions visible in magnetic resonance imaging (MRI) except for WMHs and lacunae, other medical complications, active alcohol or illicit drug use, and pregnancy.

### Clinical Evaluation

All participants underwent a comprehensive neuropsychological battery, including the following sections: (1) general cognitive ability: MMSE was used to measure general cognitive ability for each participant; (2) memory tests included auditory memory, visual memory and working memory. Chinese version of Rey’s Auditory Verbal Learning Test (RAVLT) is extensively used to assess auditory memory ability; visual memory was the Rey-Osterrieth Complex Figure Test (ROCF), and the backward Digital Span Test (B-DST) was applied to test working memory; (3) attention/executive function: Trail Making Test, part A (TMT-A); Trail Making Test, part B (TMT-B); the Stroop Color-Word Test; (4) language skills: Boston Naming Test (BNT, 30-item version) and the verbal fluency test (VFT); (5) visuospatial function: clock drawing test (CDT).

### MRI Acquisition

Magnetic resonance imaging scanning was performed on a GE Signa HDxt 3.0T scanner (General Electric Medical Systems) using an eight-channel phased-array head coil. Foam padding was used to restrict head movement, and ear plugs were used to minimize scanner noise. The parameters of the high-resolution 3D-T1 were as follows: TR = 8.3 ms, TE = 3.3 ms, flip angle = 15°, thickness = 1.0 mm, intervals = 0 mm, field of view (FOV) = 240 mm × 240 mm, matrix = 240 × 240, voxel = 1 × 1 × 1 mm^3^. WMHs and LIs were observed on T2-fluid-attenuated inversion recovery (T2-FLAIR)-weighted images. The parameters of T2-FLAIR-weighted images are TR = 8,000 ms, TE = 126 ms, TI = 1,500 ms, thickness = 5.0 mm, intervals = 1 mm, FOV = 240 mm × 240 mm, and matrix = 256 × 192.

### Data Preprocessing

Images were preprocessed, and the cortical thickness was calculated with the FreeSurfer 6.0 image analysis suite (Martinos Center for Biomedical Imaging Center, MGH^1^) (Fischl, 2012). The images were sequentially preprocessed using the following steps: intensity normalization; skull stripping (Sled et al., 1998; Segonne et al., 2004); transformation into Talairach space; segmentation of subcortical white and gray matter structures; intensity normalization to correct the non-uniformity of MR intensity, mainly caused by variations in reception coil sensitivity and gradient-driven eddy currents (Sled et al., 1998); tessellation of the gray matter/white matter boundaries; automated topology correction (Fischl et al., 2001); surface deformation following intensity gradients to optimally place the gray/white and gray/cerebrospinal fluid borders that most accurately define the transition to the other tissue class (Dale et al., 1999; Fischl and Dale, 2000); and registration to average surface space. Prior to statistical analyses, the cortical volume of each subject was presmoothed with a 15-mm, full width at half-maximum (FWHM) Gaussian kernel. The brain images were overlaid with pial and white matter surfaces to verify the quality of surface reconstruction.

The lesion probabilities of the two groups (SIVD-NC and SIVD-CI) were segmented on T2 FLAIR images by a lesion prediction algorithm (O’Neill, 2009; Egger et al., 2016) as implemented in the LST toolbox^2^ for SPM.

### Statistics

First, differences in cortical thickness among the three groups (the SIVD-NC, SIVD-CI and HC groups) were compared by one-way analysis of variance (ANOVA) followed by *post hoc* tests, with age, gender, years of education and intracranial volume (ICV) as nuisance variables. Multiple comparisons were corrected using Monte Carlo simulation correction with an initial vertex-wise threshold of *p* < 0.001 and vertex level corrected to *p* < 0.05. Second, we extracted average cortical thickness as regions of interest (ROIs) in the brain regions, and there was a significant difference in the above analysis. Then, ANOVA was conducted to examine differences in these ROIs between the SIVD-NC and SIVD-CI groups, and the false discovery rate (FDR) was used to test the significance of the *post hoc* analysis. Pearson’s correlation analyses were performed to examine the relationship between significantly different cortical thicknesses and cognitive test scores controlled for age, gender, years of education and ICV. Finally, the script written by Preacher and Hayes (2008) was used to conduct a mediation analysis using SPSS 21.0 (Chicago, IL, United States) to investigate whether a mediating variable affected the relationship between an independent variable and a dependent variable. We conducted a mediation analysis to explore whether the relationship between the WMH volume and RAVLT immediate recall scores was influenced by atrophic left MTL or precuneus. We chose the WMH volume, the thickness of left MTL (or precuneus), and the RAVLT immediate recall score (or ROCF recall score) as the independent variable, the proposed mediator and the dependent variable, respectively.

## Results

### Demographic and Cognitive Assessment

**FIGURE 1:**
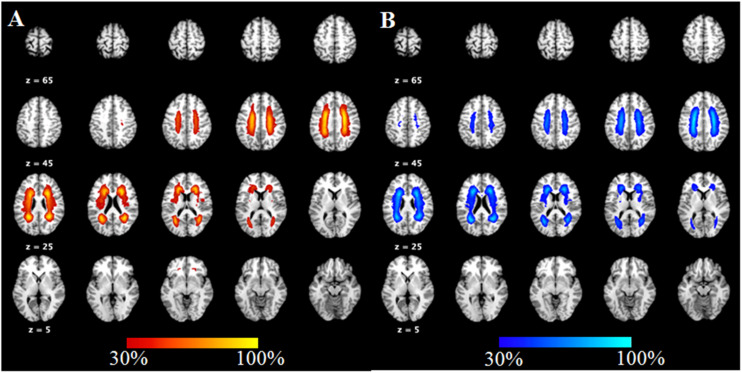
The lesion probability of white matter hyperintensities in patients with subcortical ischemic vascular disease.

Seventeen subjects were excluded because they were identified as having other nervous system diseases during MRI scanning, were incapable of completing the neuropsychological assessments, or had head movement. Thus, 67 SIVD patients without (SIVD-NC, *n* = 35) and with (SIVD-CI, *n* = 32) CI and the HC group (*n* = 36) were included in the study. The demographic and clinical features for all subjects are shown in Table 1. There were no significant differences in gender (*p* = 0.663), age (*p* = 0.714) or education years (*p* = 0.178) among these three groups. As Figure 1 shows, the lesion probability in the SIVD-NC group (Figure 1A) was similar to that in the SIVD-CI group (Figure 1B). However, the lesion distribution in the frontoparietal white matter was more extensive in the SIVD-CI group than in the SIVD-NC group.

**TABLE 1:** Demographic and clinical characteristics of subjects.

	HC	SIVD-NC	SIVD-CI	SIVD-NC vs. SIVD-CI vs. HC		SIVD-CI vs. HC	SIVD-NC vs. SIVD-CI	SIVD-NC vs. HC
				*F*/χ^2^	*p*	*p*	*p*	*p*
Gender (M/F)	19/17	21/14	19/13	0.41	0.66	0.46	0.99	0.41
Age (years)	68.22 ± 6.02	69.20 ± 5.10	69.22 ± 6.26	0.34	0.71	0.42	0.99	0.48
Education	9.47 ± 3.00	10.23 ± 2.59	9.06 ± 2.05	1.76	0.18	0.52	0.07	0.22
BMI	22.94 ± 1.77	24.36 ± 2.43	23.06 ± 2.86	3.84	0.03	0.83	0.03	0.01
HR	71.50 ± 9.68	72.21 ± 11.38	74.47 ± 10.26	0.76	0.47	0.24	0.37	0.77
Systolic blood pressure (mmHg)	129.56 ± 15.26	147.75 ± 21.67	147.75 ± 21.95	9.28	<0.001	<0.001	0.81	<0.001
Diastolic blood pressure (mmHg)	76.89 ± 9.45	82.12 ± 11.88	79.591 ± 9.49	2.24	0.11	0.28	0.32	0.04
Fasting glucose (mmol/L)	6.93 ± 1.90	6.41 ± 1.83	5.73 ± 1.18	1.37	0.26	0.69	0.12	0.23
Triglycerides (mmol/L)	1.46 ± 0.70	1.50 ± 0.86	1.55 ± 0.87	0.09	0.92	0.68	0.81	0.86
Total cholesterol (mmol/L)	5.08 ± 1.23	4.24 ± 0.79	4.49 ± 1.31	4.66	0.01	0.04	0.40	0.004
History								
Hypertension	16.67%	62.86%	68.75%	14.17	<0.001	<0.001	0.59	<0.001
Diabetes mellitus	8.33%	40.00%	18.75%	5.73	0.004	0.29	0.03	0.001
Smoking	25.00%	28.57%	28.12%	0.07	0.94	0.78	0.97	0.74
Drinking	11.11%	28.57%	21.88%	1.71	0.19	0.27	0.50	0.07
WMHs (ml)	1.10 ± 1.16	13.50 ± 3.53	16.15 ± 4.54	83.94	<0.001	<0.001	0.001	<0.001
Lacunes	0.34 ± 0.58	2.24 ± 1.73	2.48 ± 2.14	69.96	<0.001	<0.001	0.08	<0.001

*One-way ANOVA and *post hoc* analyses were used to assess differences in the continuous variables across the groups, and the χ^2^ test was used for the gender proportions. All data represent the mean ± standard deviation unless otherwise indicated. BMI, body mass index; F, female; HCs, healthy controls; HR, heart rate; M, male; SIVD-CI, subcortical ischemic vascular disease with cognitive impairment; SIVD-NC, subcortical ischemic vascular disease without cognitive impairment; WMHs, white matter hyperintensities.*

We observed significant differences in all cognitive tests among the three groups (Table 2). The SIVD-CI group performed significantly worse than the HC group or the SIVD-NC group in all cognitive tasks, whereas the SIVD-NC group showed lower scores than the HC group in the RAVLT delayed recall, TMT, VFT and Stroop Color-Word Test.

**TABLE 2:** Cognitive characteristics of the subjects.

	HC	SIVD-NC	SIVD-CI	SIVD-NC vs. SIVD-CI vs. HC		SIVD-CI vs. HC	SIVD-CI vs. SIVD-NC	SIVD-NC vs. HC
				*F*	*p*	*p*	*p*	*p*
MMSE	28.06 ± 1.14	27.83 ± 1.23	21.78 ± 2.72	53.053	<0.001	<0.001	<0.001	0.734
CDR	0	0	0.5	–	–	–	–	–
ADL	9.54 ± 3.38	2.28 ± 2.02	2.83 ± 2.77	0.579	0.562	0.370	0.979	0.345
RAVLT								
Immediate recall	7.83 ± 1.48	7.43 ± 2.27	4.03 ± 2.19	32.824	<0.001	<0.001	0.006	0.493
Delayed recall	8.94 ± 2.12	7.71 ± 2.53	3.59 ± 2.88	51.253	<0.001	<0.001	<0.001	0.047
Recognition	19.67 ± 3.55	21.17 ± 6.16	14.84 ± 7.85	9.942	<0.001	0.001	<0.001	0.296
BNT	23.75 ± 3.41	23.31 ± 4.46	18.62 ± 4.24	16.319	<0.001	<0.001	<0.001	0.651
VFT	38.25 ± 4.87	34.71 ± 6.27	24.75 ± 7.55	41.658	<0.001	<0.001	<0.001	0.019
B-DST	2.16 ± 1.44	2.16 ± 1.44	2.75 ± 0.98	8.710	<0.001	<0.001	<0.001	0.912
CDT	3.19 ± 0.67	3.14 ± 1.22	2.16 ± 1.44	38.432	<0.001	<0.001	0.001	0.849
TMT-A (s)	78.33 ± 23.60	106.65 ± 41.97	190.09 ± 83.01	38.432	<0.001	<0.001	<0.001	0.030
TMT-B (s)	187.01 ± 86.46	247.66 ± 69.80	385.17 ± 116.35	37.957	<0.001	<0.001	<0.001	0.005
ROCF								
Immediate recall	18.92 ± 5.79	17.86 ± 6.80	9.63 ± 6.90	18.079	<0.001	<0.001	<0.001	0.438
Delayed recall	15.92 ± 5.79	13.23 ± 7.76	6.63 ± 6.89	16.271	<0.001	<0.001	<0.001	0.102
Stroop Color-Word test	100.22 ± 12.13	92.46 ± 13.97	69.42 ± 19.98	34.949	<0.001	<0.001	<0.001	0.037

*One-way ANOVA and *post hoc* analyses were used to assess differences in the continuous variables across the groups. All data represent the mean ± standard deviation unless otherwise indicated. RAVLT, Rey’s Auditory Verbal Learning Test; BNT, Boston Naming Test; CDR, Clinical Dementia Rating Scale; CDT, Clock Drawing Test; HAMA, Hamilton Anxiety Rating Scale; HAMD, Hamilton Depression Rating Scale; HC, healthy controls; MMSE, Mini-Mental State Examination; B-DST, backward Digital Span Test; ROCF, Rey-Osterrieth Complex Figure Test; SIVD-CI, subcortical ischemic vascular disease with cognitive impairment; SIVD-NC, subcortical ischemic vascular disease without cognitive impairment; TMT, Trail Making Test; VFT, Verbal Fluency Test; ADL, Activity of Daily Living.*

### Cortical Thickness Differences Among the Three Groups

**FIGURE 2:**
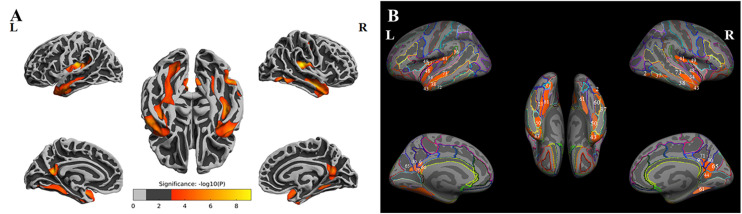
The results of one-way analysis of variance (ANOVA). **(A)** Brain regions with significant differences among the three groups based on one-way ANOVA. **(B)** Inflated view of the labeled ANOVA results.

**FIGURE 3:**
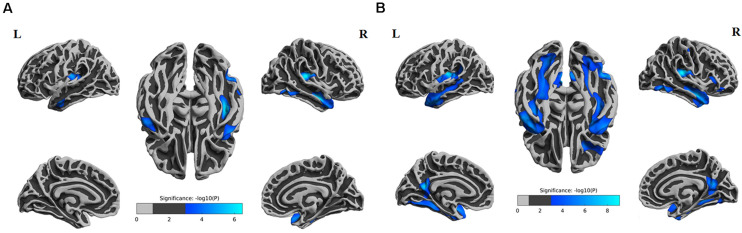
Comparison of *post hoc* results in significant brain regions between each pair of groups. **(A)** The difference in thickness between subcortical ischemic vascular disease without cognitive impairment (SIVD-NC) patients and healthy controls (HCs). Blue indicates that the thickness in SIVD-NC patients was lower than that in HCs. **(B)** The difference in thickness between the subcortical ischemic vascular disease with cognitive impairment (SIVD-CI) group and HCs. Blue indicates that the thickness in the SIVD-CI group was less than that in HCs. HCs, healthy controls; L, left; R, right.

ANOVA results showed differences in cortical thickness in the left insula, left middle and inferior temporal gyrus, left precuneus, left MTL, right insula, right superior temporal gyrus, right inferior temporal gyrus, right MTL, right postcentral gyrus and right precuneus (*p* < 0.05, Monte Carlo simulation corrected) (Table 3 and Figure 2A). We also added the figure with named labels (Figure 2B) and Table 4 to show anatomical names for ANOVA results according to FreeSurfer software templates (Destrieux et al., 2010). Then, *post hoc* pairwise comparisons were used to compare the cortical thickness changes between each pair of groups. Significantly reduced cortical thickness was found in the following brain regions in the SIVD-NC group compared to the HC group: the left insula, left middle temporal lobe, right insula, right middle temporal gyrus, and right inferior temporal gyrus (Table 3 and Figure 3A) (*p* < 0.05, Monte Carlo simulation corrected). The SIVD-CI group showed significantly decreased cortical thickness in the left insula, left precuneus, left middle and inferior temporal gyrus, left MTL, right insula, right MTL, right middle temporal gyrus, right inferior temporal gyrus, right lateral orbitofrontal cortex, right precuneus and right postcentral gyrus compared with the HC group (Table 3 and Figure 3B) (*p* < 0.05, Monte Carlo simulation corrected). There were no significant differences in cortical thickness between SIVD-NC and SIVD-CI using the Monte Carlo simulation correction.

**TABLE 3:** Brain regions with significant differences in cortical thickness among the three groups.

	Brain regions	MNI coordinate			Size	Peak *z* value
		*x*	*y*	*z*		
Group effect	L insula	–32.5	–23.2	16.9	1, 619.98	8.13
	L middle and inferior temporal lobe	–31.9	–34.4	–15.3	1, 701.12	5.02
	L precuneus	–6.9	–52.0	17	483.34	7.46
	R medial temporal lobe	31.9	–22	–25	1, 701.12	5.052
	R superior temporal	49.5	–4.6	–23.6	2, 361.26	7.642
	R precuneus	22.3	–57.5	18.6	972.60	5.921
	R insula	33.8	–28.9	14.0	840.26	9.147
	R inferior temporal	51.5	–56.8	–10.7	1, 196.14	6.761
	R postcentral gyrus	34.4	–17.4	37.3	330.28	4.640
SIVD-NC vs. HC	L middle temporal lobe	–56.7	–4.9	–28.7	447.52	–4.373
	L insula	–32.4	–23.3	15.7	344.81	–5.031
	R middle temporal lobe	49.6	–6.1	–22.8	1, 581.88	–6.164
	R insula	34.4	–29.1	13.9	563.43	–6.491
	R inferior temporal lobe	42.7	–20.3	–22.0	902.75	–6.008
	R superior temporal lobe	49.2	–3.4	–23.3	2, 994.02	–7.417
	R inferior temporal lobe	51.1	–56.8	–10.7	2, 024.44	–6.869
SIVD-CI vs. HC	L insula	–32.9	–22.3	17.6	2, 012.35	–8.608
	L middle and inferior temporal lobe	–33.1	–30.2	–16.0	3, 512.75	–7.561
	L precuneus	–6.9	–52.0	17.0	8.7.73	–7.972
	R medial temporal lobe	30	–25	–23	1, 942.85	–5.357
	R middle temporal lobe	49.2	–3.4	–23.3	2, 994.02	–7.417
	R precuneus	22.8	–58.0	18.6	1, 218.77	–6.562
	R insula	33.8	–28.9	14	1, 134.35	–9.097
	R lateral orbitofrontal gyrus	23.1	29.7	–11.0	511.58	–5.135
	R postcentral gyrus	49.2	–16.2	52.1	389.99	–4.346

*HC, healthy control; L, left; SIVD-NC, subcortical ischemic vascular disease without cognitive impairment; MNI, Montreal Neurological Institute; SIVD-CI, subcortical ischemic vascular disease with cognitive impairment, R, right.*

**TABLE 4:** List of full anatomical names for each label.

Index	Full name
2	Inferior occipital gyrus (O3) and sulcus
9	Posterior-dorsal part of the cingulate gyrus (dPCC)
10	Posterior-ventral part of the cingulate gyrus (vPCC, isthmus of the cingulate gyrus)
17	Long insular gyrus and central sulcus of the insula
21	Lateral occipito-temporal gyrus (fusiform gyrus, O4-T4)
30	Precuneus (medial part of P1)
33	Anterior transverse temporal gyrus (of Heschl)
34	Lateral aspect of the superior temporal gyrus
35	Planum polare of the superior temporal gyrus
36	Planum temporale or temporal plane of the superior temporal gyrus
37	Inferior temporal gyrus (T3)
38	Middle temporal gyrus (T2)
41	Posterior ramus (or segment) of the lateral sulcus (or fissure)
43	Temporal pole
44	Calcarine sulcus
47	Anterior segment of the circular sulcus of the insula
48	Inferior segment of the circular sulcus of the insula
49	Superior segment of the circular sulcus of the insula
50	Anterior transverse collateral sulcus
51	Posterior transverse collateral sulcus
61	Medial occipito-temporal sulcus (collateral sulcus) and lingual sulcus
65	Parieto-occipital sulcus (or fissure)
66	Pericallosal sulcus (S of corpus callosum)
71	Subparietal sulcus
72	Inferior temporal sulcus
73	Superior temporal sulcus (parallel sulcus)
74	Transverse temporal sulcus

**FIGURE 4:**
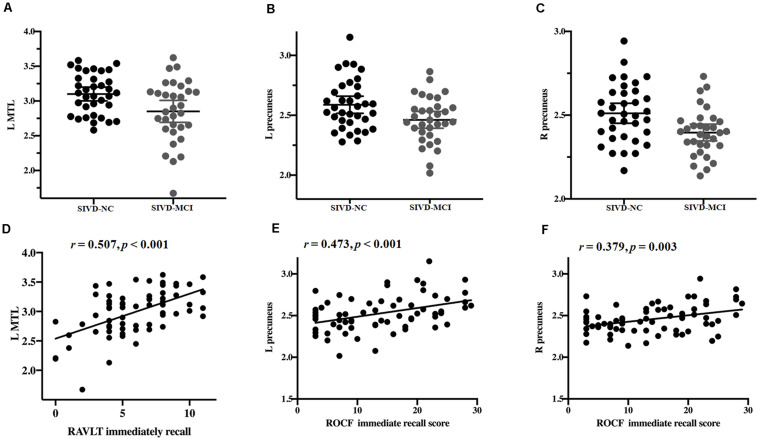
Differences in the mean thickness between the subcortical ischemic vascular disease without cognitive impairment (SIVD-NC) and with cognitive impairment (SIVD-CI) groups; correlation between cognitive scores and the mean thickness. Differences in the left medial temporal lobule (MTL) **(A)** and left **(B)** and right **(C)** precuneus between the SIVD-NC and SIVD-CI groups. **(D)** Correlations between Rey’s Auditory Verbal Learning Test (RAVLT) immediate recall and the left MTL in patients with SIVD. Correlations between left **(E)** and right **(F)** precuneus and Rey-Osterrieth complex figure test (ROCF) immediate recall scores in patients with SIVD. HC, healthy controls; L, left, R, right.

From the above results, we found that significantly decreased thickness in the bilateral insula, bilateral inferior and superior temporal gyrus in both the SIVD-NC and SIVD-CI groups compared with the HC group, while the sizes of these areas of reduced thickness in the SIVD-CI group were larger than those in the SIVD-NC group. However, reduced cortical thickness of the bilateral precuneus, MTL and right lateral orbitofrontal regions was observed only in the SIVD-CI group. Because there were no significant differences in cortical thickness between SIVD-NC and SIVD-CI using the Monte Carlo simulation correction, we extracted the cortical thicknesses of these brain regions that were significantly different according to ANOVA and then compared thicknesses between the SIVD-NC and SIVD-CI groups. We found that the SIVD-CI group, compared with the SIVD-NC group, had significantly reduced thickness in the left MTL (*p* = 0.005) (Figure 4A) and the bilateral precuneus (left: *p* = 0.005, right: *p* = 0.004) (Figures 4B,C and Table 5).

**TABLE 5:** Comparison of cortical thickness between subcortical ischemic vascular disease without and with cognitive impairment.

Brain regions	SIVD-NC	SIVD-CI	*t* value	*p* value	Adjusted *p* value
L insula	2.11 ± 0.19	2.02 ± 0.16	2.090	0.041	0.082
L middle and inferior temporal lobe	2.93 ± 0.19	2.83 ± 0.20	2.128	0.037	0.082
L precuneus	2.59 ± 0.21	2.46 ± 0.19	2.903	0.005*	0.020*
L medial temporal lobe	3.10 ± 0.29	2.85 ± 0.44	2.905	0.005*	0.020*
R superior temporal lobe	2.86 ± 0.19	2.80 ± 0.14	1.594	0.116	0.155
R precuneus	2.51 ± 0.17	2.40 ± 0.14	2.968	0.004*	0.020*
R insula	2.23 ± 0.16	2.17 ± 0.12	1.818	0.074	0.123
R inferior temporal lobe	2.84 ± 0.30	2.78 ± 0.30	0.696	0.489	0.534
R postcentral gyrus	1.79 ± 0.13	1.77 ± 0.11	0.425	0.672	0.672
R lateral orbitofrontal gyrus	2.60 ± 0.14	2.52 ± 0.18	2.158	0.035	0.082
R middle temporal lobe	2.80 ± 0.20	2.75 ± 0.14	1.109	0.272	0.326
R medial temporal lobe	3.04 ± 0.31	2.96 ± 0.32	1.721	0.082	0.123

All data represent the mean ± standard deviation unless otherwise indicated. *p* values with “*” indicate significant differences. L, left; SIVD-NC, subcortical ischemic vascular disease without cognitive impairment; SIVD-CI, subcortical ischemic vascular disease with cognitive impairment; R, right.

### Relationship Between the Alteration of Cortical Thickness and Cognitive Deficits

**FIGURE 5:**
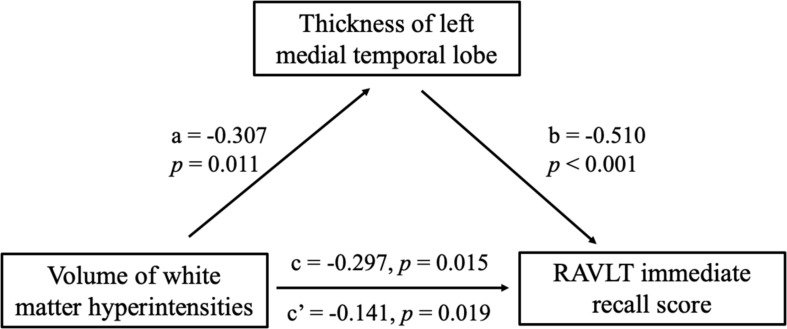
Mediation effects of the thickness of left medial temporal lobe on the relationship between the white matter hyperintensities and RAVLT immediate recall scores. RAVLT, Rey’s Auditory Verbal Learning Test.

Partial correlation was used to assess the relationship between significant differences in brain region and cognitive scores in patients with SIVD (Table 6). The results showed that the thickness of the left MTL was positively correlated with RAVLT immediate recall scores (*r* = 0.507, *p* < 0.001) (Figure 4D). The thickness of the left (*r* = 0.473, *p* < 0.001) (Figure 4E) and right (*r* = 0.379, *p* = 0.003) (Figure 4F) precuneus was correlated with the ROCF immediate recall score. The mediation analyses also revealed that the WMHs had a significant indirect effect on RAVLT immediate recall scores via the thickness of the left MTL (Figure 5). In further analysis, we found that the thickness of the left MTL was positively associated with RAVLT immediate recall scores (*r* = 0.586, *p* = 0.001) in the SIVD-CI group. The left precuneus was related to ROCF immediate recall scores in the SIVD-NC group (*r* = 0.381, *p* = 0.046) and SIVD-CI group (*r* = 0.388, *p* = 0.031). The thickness of the right precuneus was correlated with ROCF immediate recall scores in the SIVD-CI group (*r* = 0.458, *p* = 0.010).

**TABLE 6:** Correlations between the behavior scores and the regions with differences.

Behavior examination	Left medial temporal lobe		Left precuneus		Right precuneus	
	*r* value	*p* value	*r* value	*p* value	*r* value	*p* value
RAVLT immediate recall	0.507	< 0.001*	0.143	0.273	0.184	0.156
RAVLT short-delay recall	0.292	0.022	0.201	0.121	0.223	0.084
RAVLT long-delay recall	0.333	0.009	0.272	0.034	0.277	0.031
RAVLT recognition	0.147	0.257	0.166	0.201	0.238	0.064
BNT	0.035	0.790	0.066	0.612	0.247	0.055
VFT	0.266	0.038	0.316	0.013	0.220	0.089
B-DST	0.144	0.268	0.094	0.471	0.066	0.613
CDT	0.128	0.327	0.263	0.041	0.169	0.192
TMT-A (RT)	–0.005	0.971	–0.180	0.166	–0.170	0.191
TMT-B time	–0.149	0.706	–0.281	0.028	–0.267	0.037
ROCF immediate recall	0.099	0.449	0.473	< 0.001*	0.379	0.003*
ROCF delayed recall	0.052	0.689	0.230	0.074	0.118	0.363
Stroop Color-Word Test	–0.042	0.1749	–0.196	0.130	–0.055	0.672

p values with “*” indicate significant correlations. RAVLT, Rey’s Auditory Verbal Learning Test; BNT, Boston Naming Test; CDT, Clock Drawing Test; B-DST, backward Digital Span; ROCF, Rey-Osterrieth Complex Figure Test; TMT, Trail Making Test; VFT, Verbal Fluency Test.

## Discussion

In this study, we assessed alterations in cortical thickness in SIVD patients with MCI (SIVD-CI group) and without cognitive deficits (SIVD-NC group) compared to healthy senior volunteers. Both the SIVD-CI and SIVD-NC groups showed extensive thinning of the cerebral cortex, including the bilateral temporal lobe and insula, compared to the HC group, but the area of atrophy was distinctly smaller in the SIVD-NC group than in the SIVD-CI group. In addition, the SIVD-CI group exhibited significant thinning of the bilateral precuneus and left MTL compared with the SIVD-NC group. More importantly, the thickness of the left MTL was positively correlated with the RAVLT immediate recall score, and the thickness of the bilateral precuneus was related to the ROCF immediate recall score.

Our results indicated that there were extensive cognitive deficits, including episodic memory, working memory, spatial processing, and language, processing speed and executive function, in SIVD-CI patients, which is consistent with results from previous studies. Several cross-sectional studies (O’Brien et al., 2003; Gorelick et al., 2011; Scherr et al., 2014) found that patients with SIVD showed significant differences in test scores measuring verbal fluency, verbal memory, speed of cognitive processing, and divided attention when compared with healthy elderly volunteers. Zeng et al. (2019) suggested that higher WMH load scores were associated with worse performance in several cognitive domains, most notably in memory. A longitudinal study (Wang et al., 2020) recently showed that an increased WMH volume was linked to a faster cognitive decline at baseline and follow-up.

We have demonstrated that patients with SIVD showed cortical atrophy in the bilateral insula, temporal lobe, precuneus, MTL and right postcentral gyrus. Previous studies (Tuladhar et al., 2015; Dickie et al., 2016; Lambert et al., 2016) have demonstrated that extensive white matter lesions may cause atrophy in remote cortical areas, and a negative correlation was found between the volume or number of WMHs and cortical volume or thickness. Tuladhar et al. (2015) indicated that a higher WMH load was associated with lower cortical thickness in frontotemporal regions. (Dickie et al., 2016) performed a cross-sectional study and found associations between WMH volume and cortical thickness within and surrounding the Sylvian fissure. However, they did not find any changes between changes in WMH volume and changes in cortical thickness in a subsequent longitudinal study. Lambert et al. (2015) showed that the rate of white matter hyperintensity progression is associated with increases in cortical gray matter atrophy rates in the mediofrontal, orbitofrontal, parietal and occipital regions. This cortical atrophy may be related to disrupted connectivity between white matter and cortical gray matter due to white matter lesions, which would secondarily cause atrophy of the distal cortex.

Our results also showed significantly decreased thickness in the bilateral insula and bilateral inferior and superior temporal gyrus in both the SIVD-NC and SIVD-CI groups compared with the HC group, whereas reduced thicknesses in the bilateral precuneus, MTL and right lateral orbitofrontal cortex were observed only in the SIVD-CI group. Furthermore, compared to the SIVD-NC group, the SIVD-CI group showed significantly reduced thicknesses in the bilateral precuneus and left MTL. Grau-Olivares et al. (2010) compared changes in gray matter volume between baseline and follow-up in lacunar stroke patients with MCI. Their results showed that gray matter volume losses occurred mainly in the frontal and temporal cortices as well as the pons, cerebellum and caudate nucleus. Although both their study and ours found atrophy in the temporal lobe, there were some differences between the two studies. The reasons for the discrepancy may be the different indices (gray matter volumes in Grau-Olivares et al. and cortical thickness in this study) and analysis methods (longitudinal study in Grau-Olivares et al. and cross-sectional study in our study). Lei et al. (2016) found that patients with vascular MCI exhibited significantly reduced gray matter volume in the bilateral DLPFC, the orbital portion of the SFG, the left supplemental motor area, and the bilateral PCC compared to a healthy elderly population. However, it has not yet been reported whether there is a distinct difference in cortical atrophy between SIVD without and with MCI. Our results suggested that extensive cortical atrophy occurred in patients with SIVD and that the range of atrophy was wider in SIVD with MCI than in SIVD without MCI. Furthermore, specific regional atrophy in the cortex might lead to cognitive deficits in patients with SIVD.

In this study, we found that the thickness of the left MTL was related to RAVLT immediate recall scores in the patients with SIVD. The MTL includes the hippocampus, dentate gyrus, subicular complex, and adjacent perirhinal, entorhinal, and parahippocampal cortices. The hippocampal region includes the CA, dentate gyrus, fimbria, molecular-layer, hippocampal fissure, subiculum, parasubiculum, presubiculum, and hippocampal amygdalar transition area (Squire et al., 2007). The cortex of the medial temporal cortex plays a vital role in episodic memory function and includes a system of anatomically related structures that consists of the hippocampal region and the adjacent perirhinal, entorhinal, and parahippocampal cortices (Squire et al., 2004). It has been reported that district atrophy occurs in the MTL in patients with Alzheimer’s disease or patients with vascular dementia. MTL atrophy revealed significant correlations with most of the cognitive function tests that we examined, including verbal memory, orientation, and spatial ability (Jokinen et al., 2012; Arba et al., 2017). Bednarz-Misa et al. (2020) suggested that decreased paraoxonase 1 was more associated with vascular involvement and the severity of brain atrophy or ischemia. In addition, the WMHs may have a direct or indirect effect on RAVLT immediate recall scores in SIVD patients via decreased thickness of the left MTL, suggesting that WMHs affect the thickness of the left MTL, resulting in reduced RAVLT immediate recall in individuals with SIVD. Our results indicated that the thickness of the MTL was positively correlated with the verbal recall score in patients with CI, which suggested that reduced thickness of the MTL might be associated with memory impairment in patients with SIVD.

The precuneus and adjacent PCC are considered to play a pivotal role in the default-mode cortical network. The findings from functional imaging in healthy subjects suggested that the precuneus has extensively/widely connected cortical and subcortical structures. The precuneus may subserve a variety of behavioral functions because it is a major association area (Cavanna and Trimble, 2006). Previous studies suggested that precuneus and PCC play a role in visuospatial imagery and spatial memory. The subjects took the ROCF test (Pelati et al., 2011), which is a commonly used neuropsychological test that evaluates spatial memory ability at the recall stage. We found thinning of the bilateral precuneus in patients with SIVD, especially those with CI, and the thickness of the bilateral precuneus was related to ROCF immediate recall scores. The results were similar to previous results (Lei et al., 2016) and suggested that it might be one of the reasons for decreased spatial memory in SIVD patients.

Our study had some limitations. First, we did not divide SIVD patients with CI into MCI and dementia in this study. We found that a specific pattern of cortical atrophy is related to cognitive deficits in the CI in patients with SIVD in this cross-sectional study. However, it is necessary to explore the changes in cortical structure from SIVD without cognitive deficits to MCI to dementia in future studies. It is difficult for researchers to confirm whether the difference in cortical atrophy between patients with and without MCI is related to the duration of disease in patients with SIVD in the cross-sectional study because the majority of patients with SIVD were found by accident. Therefore, it is necessary to longitudinally track these patients to further confirm whether cognitive decline and cortical atrophy will gradually progress over time. Second, CI in SIVD patients was identified based on MMSE and CDR scores, not a comprehensive neuropsychological assessment. Although our results confirmed that atrophy of the MTL was associated with memory deficits, we did not conduct a more detailed analysis of the hippocampal subfields. The aim of the study was to identify specific cortical atrophy in SIVD patients with MCI and its association with cognitive deficits. In a future study, we will further clarify the relationship between volume changes in subfields in the hippocampus and different types of memory impairment. Third, microbleeds have been observed in the context of white matter abnormalities, lacunar stroke and hypertension. However, we did not use paramagnetic sequences (e.g., susceptibility-weighted imaging) to detect microbleeds in this study. In follow-up studies, we will further evaluate how microbleeds affect cognitive function in the SIVD patients. Blanco-Rojas et al. (2013) suggested that the effects of WMHs and lacunar infarction on cognitive function differ in the early stage of cerebral small vessel disease. A limitation in the study was that the potential role of WMHs, microbleeds, and lacunae in CI of the patients was not assessed. The differences between the subtypes of cerebral small vessel disease will be explored in future studies. Finally, we did not measure tau and amyloid statuses, which are important indicators for Alzheimer’s disease. Considering that SIVD is related to vascular risk factors, we tested some indicators such as glucose and cholesterol, which are associated with vascular diseases.

This study observed extensive thinning in the cerebral cortex in patients with SIVD. Moreover, we found that the specific atrophied regions included the left MTL and bilateral precuneus in SIVD patients with CI, and the thickness of these regions was associated with episodic memory in SIVD patients. Our results provide potential imaging markers to predict early stage cognitive decline and improve the understanding of MCI in patients with SIVD from the perspective of pathophysiology.

## Data Availability Statement

The original contributions presented in the study are included in the article/supplementary material, further inquiries can be directed to the corresponding authors.

## Ethics Statement

This study was approved by the research ethics committees of The First Affiliated Hospital of Chongqing Medical University. Written informed consent was obtained from each participant. The patients/participants provided their written informed consent to participate in this study.

## Author Contributions

LC and TL contributed equally to the experiments, data analysis, and writing and revising the manuscript. JS was responsible for the data analysis and drafting of the manuscript. RC contributed to the data collection and data analysis. KW contributed to performing the experiments and data analysis. XL and MTL contributed to the data collection. All authors contributed to the article and approved the submitted version.

## Conflict of Interest

The authors declare that the research was conducted in the absence of any commercial or financial relationships that could be construed as a potential conflict of interest.

## Footnotes

1 http://surfer.nmr.mgh.harvard.edu/

2 www.statistical-modelling.de/lst.html
